# Supplementary material for: DMRforPairs: identifying Differentially Methylated Regions between unique samples using array based methylation profiles
Source: BMC Bioinformatics. 2014 May 15;15:141. doi: 10.1186/1471-2105-15-141 (PMC4046028; doi:10.1186/1471-2105-15-141)
Supplement: Additional file 2 — DMRforPairs output for the comparison of A431-MCF7 and NA17018-NA17105. Please start from the HTML files in each folder. Available via the BMC Bioinformatics website. [file 1471-2105-15-141-S2.zip › 1394847754114233_MOESM2_ESM/A431_MCF7/figures/34171.pdf]

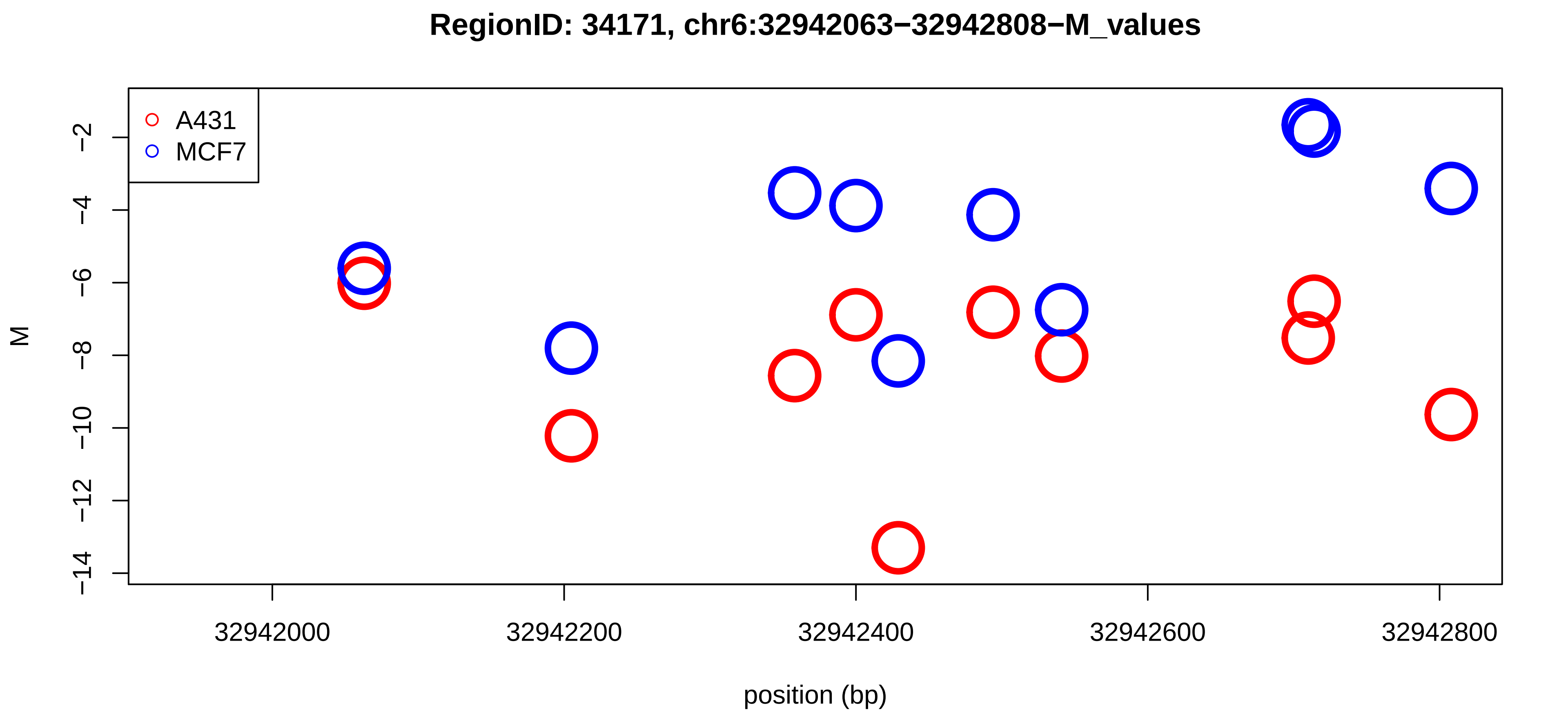
RegionID: 34171, chr6:32942063−32942808−M_values
A431
MCF7
−2
−4
−6
−8
−10
−12
−14
M
32942000
32942200
32942400
32942600
32942800
position (bp)

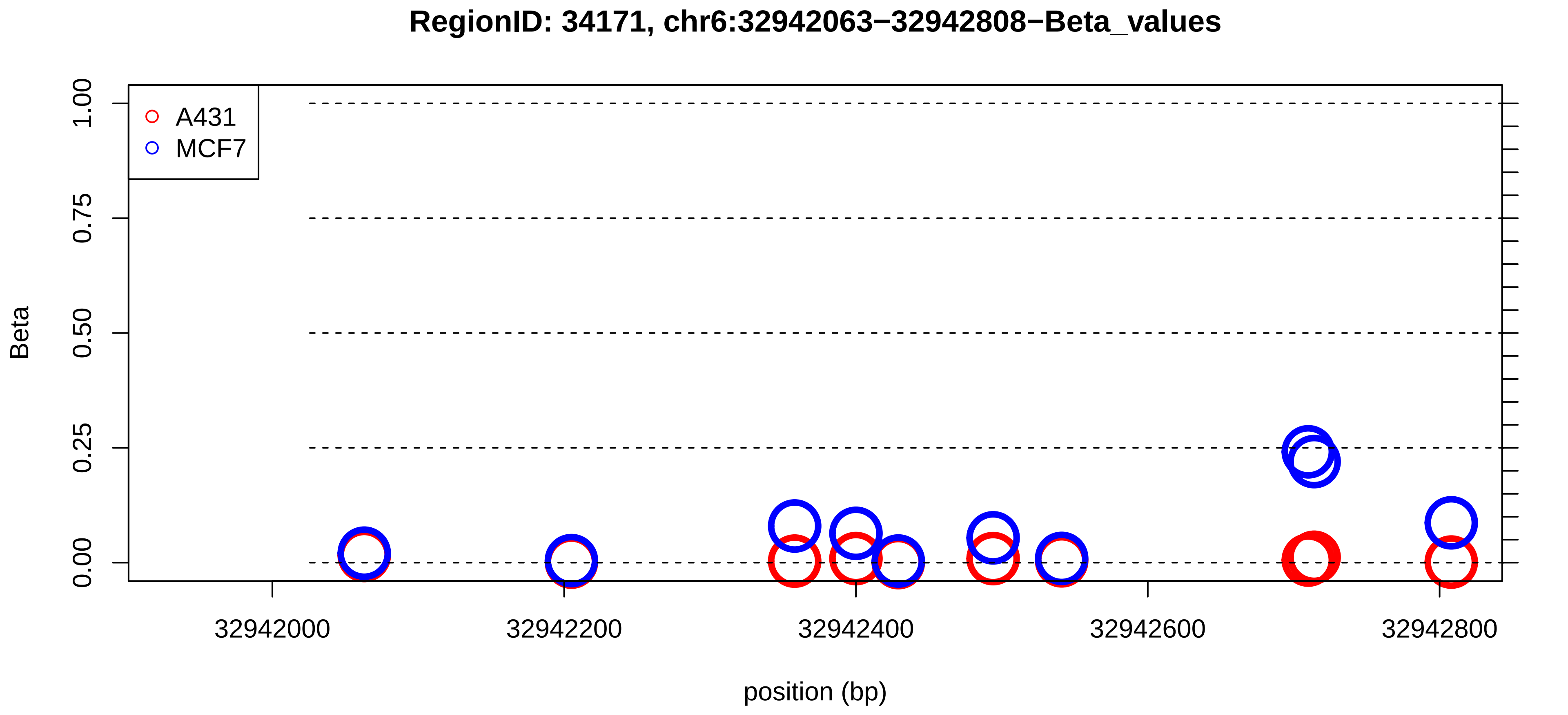
RegionID: 34171, chr6:32942063−32942808−Beta_values
A431
MCF7
1.00
0.75
0.50
0.25
0.00
Beta
32942000
32942200
32942400
32942600
32942800
position (bp)

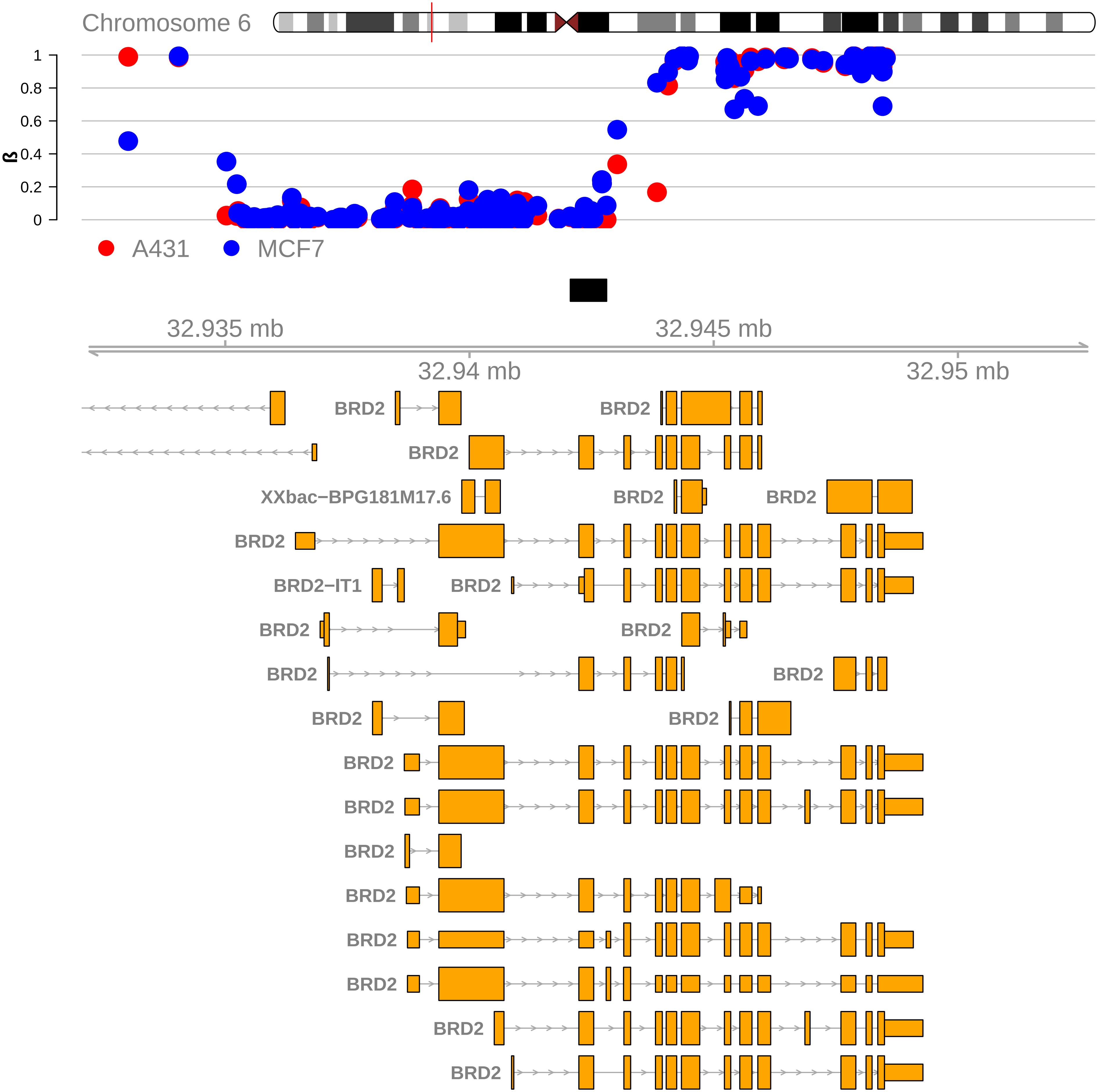

Chromosome 6
ß
1
0.8
0.6
0.4
0.2
0
A431
MCF7
32.935 mb
32.945 mb
32.94 mb
32.95 mb
BRD2
BRD2-IT1
XXbac-BPG181M17.6
